# Entzündliche Veränderungen des Hüftgelenks

**DOI:** 10.1007/s00117-021-00811-9

**Published:** 2021-02-11

**Authors:** Claudia Weidekamm, James Teh

**Affiliations:** 1 Universitätsklinik für Radiologie und Nuklearmedizin, Klinische Abteilung für Neuroradiologie und Muskuloskelettale Radiologie, Medizinische Universität Wien, Währinger Gürtel 18–20, A-1090 Wien, Österreich; 2 Department of Radiology, Nuffield Orthopaedic Centre, Oxford University Hospitals NHS Trust, Windmill Road, Headington, OX3 7LD Oxford, Großbritannien

**Keywords:** Hüftschmerz, Magnetresonanztomographie, Entzündung, Diagnose, Synovitis, Hip pain, Magnetic resonance imaging, Inflammation, Diagnosis, Synovitis

## Abstract

Die Osteoarthrose ist die häufigste Ursache für den Hüftschmerz des Erwachsenen. Daher wird anderen Ursachen wie z. B. Entzündungen weniger Beachtung für den Gelenkschmerz in der Erstdiagnose geschenkt. Dieser Artikel gibt eine Übersicht von unterschiedlichen rheumatologischen Erkrankungen der Hüfte und deren Interpretation in der Bildgebung. Die Vor- und Nachteile der einzelnen bildgebenden Verfahren werden anhand der pathologischen Befunde für die rheumatologischen Erkrankungen erläutert.

## Abstract (en)

Most cases of hip pain in adults are related to osteoarthrosis, thus other causes, e.g. inflammation, are often not adequately considered for the joint pain in the initial diagnosis. This review article describes the various rheumatic diseases of the hip in the context of diagnostic image interpretation. The advantages and disadvantages of the individual imaging modalities are discussed against the background of pathological findings of rheumatological diseases of the hip.

## Lernziele

Nach Absolvieren dieser Fortbildungseinheit …

- verstehen Sie die Zusammenhänge zwischen den morphologischen Veränderungen in der Bildgebung und der Prognose für die Hüftgelenkentzündung.
- erkennen Sie die typischen Zeichen der rheumatischen Entzündung.
- können Sie die rheumatologischen Erkrankungen unter Berücksichtigung der Klinik, des Alters des Patienten und der Laborparameter besser einordnen.
- können Sie den Kliniker unterstützen, die richtige Bildgebung für die entsprechende Fragestellung auszuwählen.

## Einleitung

Die Entzündung wird nur selten initial in der Differenzialdiagnose für den Hüftschmerz in Betracht gezogen, da in den meisten Fällen die Degeneration der Hüfte mit einem ähnlichen klinischen Erscheinungsbild assoziiert ist. Das fehlende Bewusstsein der entzündlichen Hüfte als Ursache für die Beschwerden führt zu einer Verschleppung der Diagnose und kann somit irreversible Gelenkveränderungen hervorrufen. Der Hüftschmerz ist unspezifisch und kann in die Leiste, ins Gesäß oder entlang des Beins ausstrahlen. Die klinische Untersuchung der Hüfte ist durch die anatomisch tiefe Lage unterhalb der Muskeln sowie durch andere Pathologien mit ähnlichem klinischen Erscheinungsbild wie Hernien, Sakroiliitis, Radikulopathien oder Tendinosen der ischiokruralen Muskulatur erschwert.

Zur Basisabklärung des Hüftschmerzes in der Bildgebung dient üblicherweise die Projektionsradiographie. Allerdings kann diese im Frühstadium der Entzündung unauffällig sein, was zu einer Verschleppung der Diagnose des entzündlichen Hüftschmerzes führt. Daher ist es von großer Bedeutung, im sog. **präradiographischen Stadium**

Präradiographisches Stadium

mit weiteren bildgebenden Modalitäten wie Sonographie, Magnetresonanztomographie (MRT), Positronenemissionstomographie (PET) oder Szintigraphie die Frühzeichen der Entzündung wie Synovitis oder Knochenmarködem (KMÖ) als Ursache des Schmerzes abzuklären.

Der Patient hat eine bessere Prognose, wenn die korrekte Diagnose früh gestellt und rasch eine effiziente Therapie eingeleitet werden kann. Adäquate entzündungshemmende Medikamente können irreversible Schäden wie Knorpel- und Knochendestruktionen vermeiden oder aufhalten.

Der erste Teil des CME-Artikels fasst die notwendigen Kenntnisse für den entzündlichen Hüftschmerz und die radiologische Interpretation zusammen.

## Indikation zur Bildgebung

Eine sofortige Indikation zur Bildgebung sollte nach erheblichem Trauma oder Verdacht auf Infektion oder **avaskuläre Nekrose**

Avaskuläre Nekrose

erfolgen, da hier die Gefahr eines bleibenden Gelenkschadens besteht, wenn keine sofortige Therapie erfolgt. Bei Patienten unter Kortisontherapie sollte die Bildgebung bei Hüftschmerzen großzügig eingesetzt werden, da ein erhöhtes Risiko für Knochenmarkinfarkte und osteoporotische Frakturen vorliegt.

Im Folgenden werden die Vor- und Nachteile der einzelnen Bildgebungsmodalitäten ausgeführt, um als Grundlage für die Entscheidung zu dienen, welches Verfahren vorzugsweise zu welchem Zeitpunkt und bei welchem Szenario eingesetzt werden sollte.

## Bedeutung der einzelnen bildgebenden Modalitäten

### Projektionsradiographie

Die Empfehlungen der European Society of Musculoskeletal Radiology (ESSR; [1]) und der European League Against Rheumatism (EULAR; [2]) für die Bildgebung schlagen die Projektionsradiographie als initiale Bildgebung für die Abklärung der Hüfte vor. Neben der axialen Hüftaufnahme ist die Beckenübersicht hilfreich, um die Diagnose einzuschränken, wenn beispielsweise zusätzlich **Enthesopathien**

Enthesopathien

am Beckenring oder eine Beteiligung der **Sakroiliakalgelenke**

**Figure 1:**
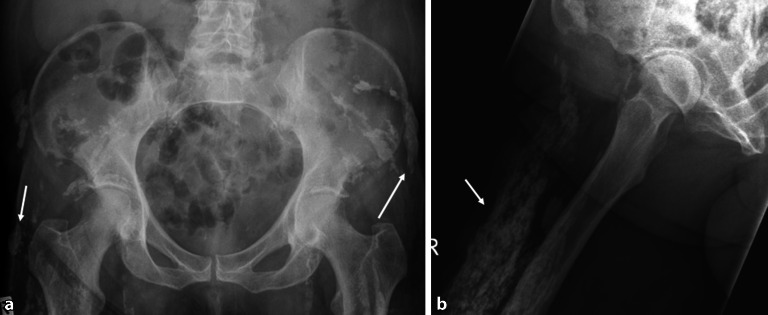

Sakroiliakalgelenke

(SIG) vorliegen, die mit der axialen Spondyloarthritis vereinbar sind. Irreversible Pathologien wie Sklerose, Knochenproliferation, Erosionen, Deformitäten des Femurkopfes, Gelenkspaltverschmälerungen und Weichteilverkalkungen sind üblicherweise in der Projektionsradiographie erkennbar und zählen zu den strukturellen Veränderungen (Abb. 1a, b).

#### Merke

Strukturelle Veränderungen sind erst im fortgeschrittenen Stadium der Erkrankung nachweisbar.

Die nichtradiographischen Veränderungen (Veränderungen, die nicht direkt in der Projektionsradiographie erkennbar sind) sind häufig frühe Läsionen, die die aktive Entzündung widerspiegeln. Zu diesen zählt der **Gelenkerguss**

Gelenkerguss,

der indirekt als Erweiterung des Gelenkspalts erkennbar ist, oder die **periartikuläre Weichteilschwellung**

Periartikuläre Weichteilschwellung,

die ein Zeichen der Synovitis sein kann. Allerdings ist hier anzumerken, dass die Projektionsradiographie nicht für den Nachweis von frühentzündlichen Veränderungen geeignet ist.

Für häufige rheumatologische Erkrankungen wurden radiographische Scoring-Systeme entwickelt, um die Wahrscheinlichkeit der Erkrankung zu ermitteln. Für die Spondyloarthritis kann der **Bath Ankylosing Radiology Hip Index (BASRI-h)**

**Figure 2:**
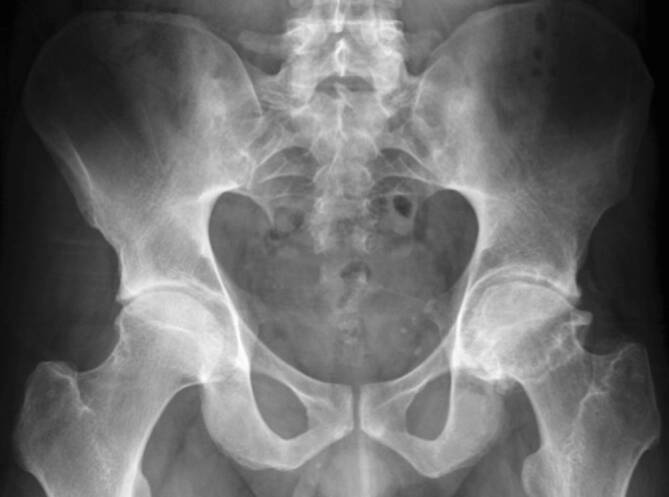

Bath Ankylosing Radiology Hip Index (BASRI-h)

hilfreich sein, um die Diagnose der ankylosierenden Spondylitis (AS) zu manifestieren [3]. Zu den radiologischen Zeichen der Hüftgelenkveränderungen zählen eine gleichmäßige Gelenkspaltverschmälerung, große subchondrale Erosionen und eine Protrusio acetabulae (Abb. 2).

### Sonographie

**Figure 3:**
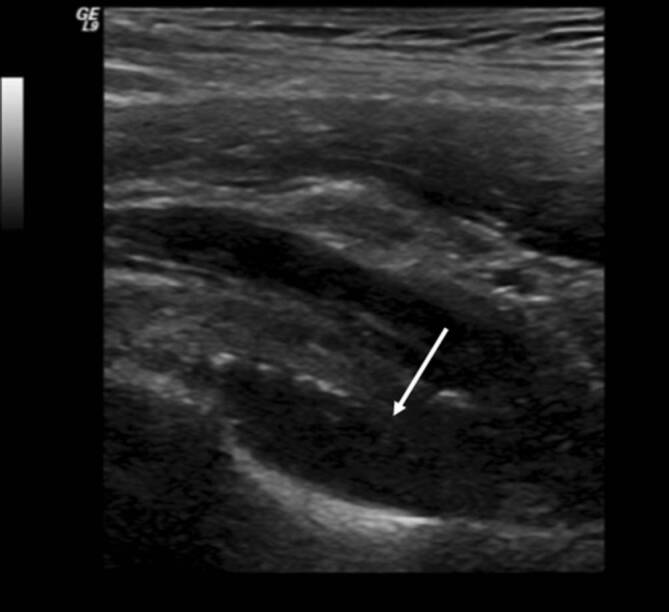

Die ESSR empfiehlt, dass die Sonographie der entzündlichen Hüfte in Rückenlage des Patienten in schrägem Längsschnitt entlang des Oberschenkelhalses durchgeführt wird, um einen Gelenkerguss im vorderen Rezessus und eine verdickte Gelenkkapsel als Ausdruck einer Synovitis darzustellen ([4]; Abb. 3). Die Synovitis verursacht vorzugsweise an der Kapselinsertion intraartikuläre Erosionen. Diese werden auch **„bare areas“**

„Bare areas“

genannt, da diese Gelenkanteile nicht von Knorpel überzogen sind. Mehrere Studien [5] haben gezeigt, dass das Vorliegen und die Progression der Erosionen mit dem Ausmaß der Krankheitsaktivität korrelieren. Die vaskularisierte aktive Synovitis wird in der Sonographie mittels Farbdoppler- oder Powerdoppleraktivität dargestellt und kann quantifiziert werden. Die Sonographie unterstützt die Aspiration von Gelenkergüssen, was die Gewinnung von selbst geringen Flüssigkeitsmengen aus dem Gelenk, z. B. für Mikrokulturen zum Nachweis einer Infektion, ermöglicht. Unter sonographischer Kontrolle wird die Komplikationsrate im Rahmen einer Gelenkpunktion (z. B. Gefäßverletzungen der Arteria circumflexa) niedrig gehalten.

### Magnetresonanztomographie

**Figure 4:**
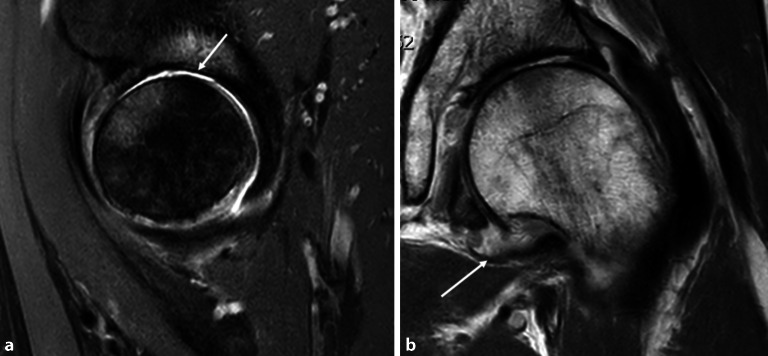

Im Studienvergleich zeigte sich, dass die MRT der klinischen Untersuchung und der Projektionsradiographie in der Diagnose der entzündlichen Hüfte überlegen ist [6]. Die MRT ermöglicht eine direkte Darstellung von Knochenmarkveränderungen und intraartikulären Strukturen, die nicht mit der Sonographie beurteilt werden können (Abb. 4 und 5). Die MRT ist daher die bildgebende Methode der Wahl, um eine frühe Entzündung der Hüfte nachzuweisen. Die intraartikuläre Kontrastmittel(KM)-Gabe ist hilfreich für den Ausschluss von Labrum- oder Knorpelläsionen, wird generell aber nicht bei Fragestellungen nach entzündlichen Veränderungen empfohlen, da Gelenkergüsse und **Synovitis**

**Figure 5:**
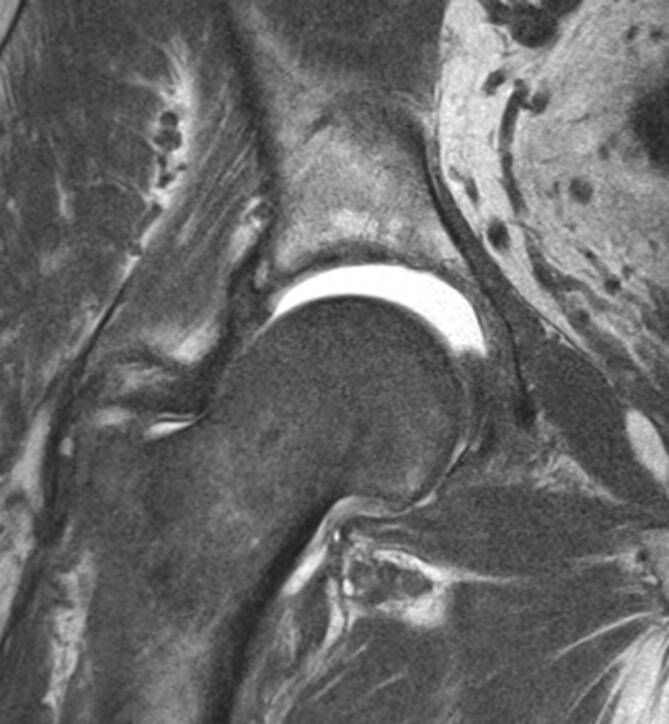

Synovitis

maskiert werden können (Abb. 5). Dagegen ist die intravenöse KM-Gabe für den Nachweis und die Bestimmung des Ausmaßes von Synovitis und **synovialen Tumoren**

Synoviale Tumoren

von Vorteil. Dynamische KM-Kurven können die Entzündungsaktivität beurteilen und möglicherweise als prognostischer Faktor eingesetzt werden. Dies wird allerdings derzeit nur für wissenschaftliche Studien verwendet und ist üblicherweise nicht im routinemäßigen Hüft-MRT-Protokoll vorgesehen. Das Arthritis Subcommittee der ESSR empfiehlt ein MRT-Protokoll, wie in Tab. 1 angeführt [7]. Die MRT spielt nicht nur in der Diagnose eine Schlüsselrolle, sondern hat auch bei der Beurteilung des Therapieansprechens und beim Nachweis von Komplikationen der Therapie einen hohen Stellenwert. Die Darstellung der SIG in der MRT-Untersuchung ist in manchen Fällen extrem hilfreich, um die Verdachtsdiagnose einer Spondylarthritis mit entzündlicher Hüftbeteiligung zu bestätigen, falls gleichzeitig eine Sakroiliitis vorliegt.

**Table 1:** 

Koronare STIR/TIRM, T2-fs oder PD-fs
Koronare T1
Axiale STIR/TIRM, T2-fs oder PD-fs
Axiale T1 (dünnschichtige Gradientenechosequenz für Erosionen)
Axiale/koronare Post-contrast-T1 oder T1-fs (falls eine weitere Abklärung einer fraglichen Synovitis, Enthesitis oder Osteitis verlangt ist)

*STIR* „short tau inversion recovery“, *TIRM* „turbo inversion recovery magnitude“, *fs* „fat saturated“, *PD* protonendichtegewichtete Sequenz

### Computertomographie

**Figure 6:**
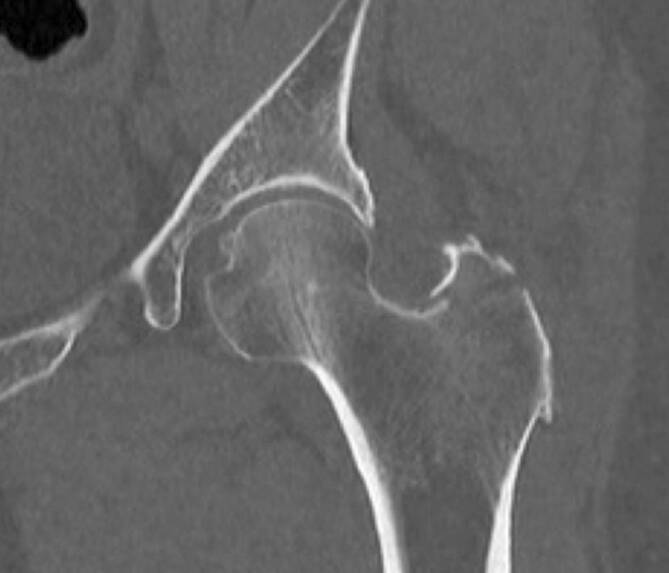

Die Computertomographie (CT) stellt freie Gelenkkörper, Erosionen, subchondrale Sklerose und Osteophyten sowie Frakturen in hoher Auflösung dar (Abb. 6). Multiplanare und 3‑D(dreidimensionale)-Reformatierungen sollten routinemäßig eingesetzt werden und unterstützen die chirurgische Planung. Der größte Nachteil der CT ist die **Strahlenexposition**

Strahlenexposition,

die besonders bei Kindern eine entscheidende Rolle spielt. Durch modernere Geräte und spezielle Protokolle kann die Strahlenexposition jedoch reduziert werden. Im Vergleich zur MRT und zur Sonographie hat die CT einen schlechteren Weichteilkontrast, weshalb Entzündungen in nativen Untersuchungen ohne KM häufig übersehen werden. Die PET-CT kombiniert die funktionelle Aktivität eines radioaktiven Tracers wie z. B. Fluordesoxyglukose mit der guten detaillierten anatomischen Darstellung in der CT und ermöglicht daher eine Beurteilung von Weichteil- und Knochenentzündung. Nachteile der PET-CT sind die Strahlenexposition und eine geringe Verfügbarkeit in den meisten Institutionen. Die **Dual-Energy-CT**

Dual-Energy-CT

hat einen wichtigen Stellenwert in der Unterscheidung zwischen Gicht und Pseudogicht (Chondrokalzinose [„calcium pyrophosphate dehydrate crystal deposition disease“, CPPD]). Die Technik der Dual-Energy-CT beruht auf der Verwendung zweier verschiedener Energiespektren, die die Differenzierung von unterschiedlichen Materialien/Strukturen ermöglicht. Bei der Hüfte spielt dies allerdings eine untergeordnete Rolle, da diese Entitäten nur selten auftreten [8].

## MRT-Protokolle: Welche Sequenzen sollen wir verwenden?

Das empfohlene Protokoll der ESSR-Arthritis-Arbeitsgruppe für rheumatologische Erkrankungen ist in Tab. 1 angeführt [7]. Die flüssigkeitssensitiven Sequenzen wie STIR/TIRM („short tau inversion recovery“/„turbo inversion recovery magnitude“) oder fettunterdrückte („fat saturated“, fs) protonendichtegewichtete (PD-) Sequenzen sind geeignet, um KMÖ, Kapselödem oder Gelenkergüsse zu beurteilen (Abb. 4). Die **STIR-Sequenz**

STIR-Sequenz

wird gegenüber der PD-fs-Sequenz bevorzugt, da sie robuster ist und verlässlicher das KMÖ darstellt, was ein entscheidendes Kriterium für entzündliche Erkrankungen ist. Allerdings ist die STIR-Sequenz bei der Bildauflösung und der Darstellung des Knorpels der **PD-Sequenz**

PD-Sequenz

unterlegen. Ins MRT-Protokoll sollte zumindest eine STIR-Sequenz, bevorzugt in koronarer Ebene, eingeschlossen werden. Die laterale Hüfte für Enthesitis der Abduktoren und für diverse Bursitiden (peritrochantäre Bursa, Bursa subgluteus medius, Iliopsoas-Bursa oder ischiogluteale Bursa) wird in den axialen und koronaren STIR- oder PD-fs-Sequenzen beurteilt. Die koronare T1-gewichtete (T1w‑) Sequenz liefert Informationen über die anatomischen Verhältnisse wie Position des Hüftkopfes, Normvarianten oder ossäre Anbauten. Das Knochenmark sowie subchondrale Frakturen werden am zuverlässigsten in der T1w-Sequenz dargestellt (Abb. 4 und 5). Eine dünne Schichtdicke von 1 mm oder weniger weist eine höhere Auflösung für den Nachweis von Erosionen auf. Aus isotropen Voxeln einer 3‑D-Sequenz können beliebige Reformationen in jeglicher Ebene (multiplanar) angefertigt werden. KM-Sequenzen mit Fettunterdrückung werden für den Nachweis von aktiver Synovitis empfohlen, da der Kontrast zwischen den anreichernden entzündlichen Strukturen und dem umgebenden Gewebe (zumeist Fettgewebe) verbessert wird. Die intraartikuläre KM-Gabe führt zu einer Distension des Gelenks und somit zu einer besseren Beurteilung von Labrum und Knorpelschäden. Die Verwendung einer zusätzlichen Traktion hat in den letzten Jahren zunehmendes Interesse gefunden. Eine spezielle MRT-taugliche Vorrichtung mit variablen Gewichten bewirkt einen für den Patienten noch tolerablen Zug an der unteren Extremität. Intraartikuläres KM umspült **Delaminationsdefekte**

Delaminationsdefekte

des Knorpels, und bei gleichzeitiger Traktion des Femurkopfes von der Pfanne verbessert sich die Beurteilung des femuralen und azetabulären Knorpelüberzugs entscheidend, da das KM die beiden Knorpelschichten voneinander trennt (Abb. 5). Dadurch sind selbst Delaminationsdefekte des Knorpels erkennbar, die sonst durch die aufeinanderliegenden Knorpelschichten maskiert werden können [9]. Auch der Nachweis von freien zentral gelegenen Gelenkkörpern wird durch die Traktion verbessert [10].

## Stellenwert der MRT für die Hüftentzündung

Sowohl die MRT als auch die Sonographie spielen eine zunehmende Rolle bei der Untersuchung von Polyarthritispatienten. Beide Verfahren sind sensitiver im Nachweis der Synovitis als die Projektionsradiographie [11]. Das KMÖ gilt als Frühzeichen für Erosionen und wird am besten in der MRT nachgewiesen (Abb. 6). Der frühe Nachweis von entzündlichen Veränderungen ermöglicht den frühzeitigen gezielten Einsatz von Medikamenten und kann irreversible Schäden verhindern. Die MRT ist das bildgebende Verfahren der Wahl für diagnostisch unklare Fälle oder für Patienten, bei denen die klinische Untersuchung erschwert ist.

Der **DAS(Disease Activity Score)-28**

DAS-28

wird als klinischer Parameter für die Beurteilung der rheumatoiden Arthritis (RA) herbeigezogen und berücksichtigt 28 Gelenke, wobei allerdings die Hüftgelenke nicht eingeschlossen sind. Somit ist es nicht überraschend, dass keine Korrelation zwischen DAS-28 und der Hüftentzündung vorliegt. Dies trifft ebenso für die juvenile rheumatische Arthritis zu. Nistala et al. konnten keine Korrelation zwischen den klinischen Scores und den Laborwerten in der MRT herstellen, was die hohe Sensitivität der MRT für die Synovitis unterstreicht [12].

## Hüftentzündung: typische Zeichen in der Bildgebung

Repräsentativ für das Vorliegen einer akuten Entzündung ist der Nachweis von (Abb. 4, 5 und 7):

- KMÖ,
- Gelenkerguss,
- pathologische synoviale KM-Anreicherung
- Enthesitis.

Zu den chronischen Veränderungen zählen (Abb. 2):

- subchondrale Sklerose,
- diffuse Gelenkspaltverschmälerung,
- Ankylose,
- Erosionen.

Akute und chronische Veränderungen können bei etwa einem Drittel der Patienten parallel auftreten [6].

**Figure 7:**
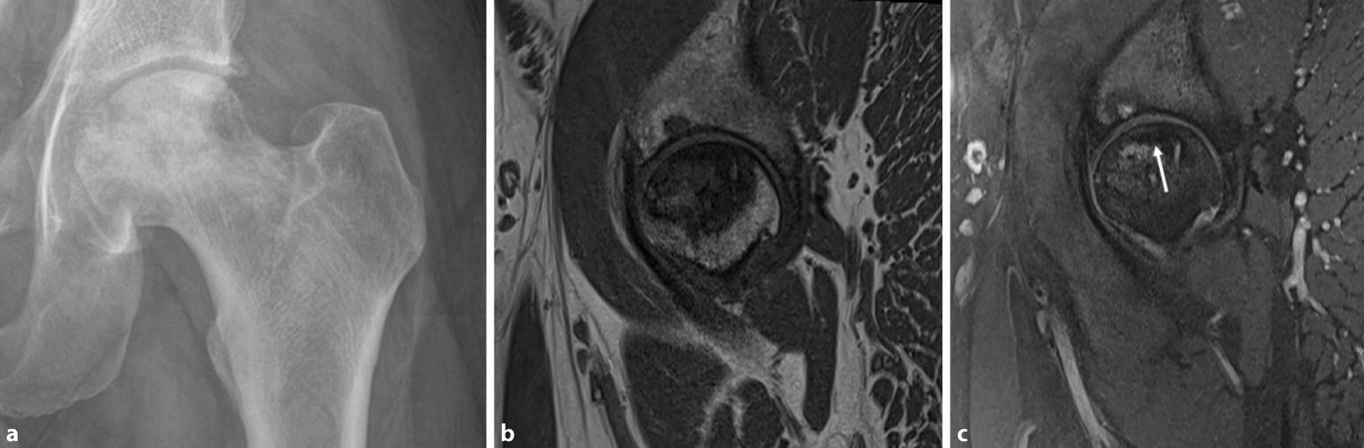

### Knochenmarködem

Das subchondrale KMÖ wird als stärkster Prädiktor für Erosionen betrachtet. In der MRT erscheint das KMÖ auf den STIR- oder den T2w-fs-Sequenzen hell und unregelmäßig begrenzt dunkel auf den T1w-Sequenzen (Abb. 6). Das histologische Korrelat für das hyperintense T2-Signal des KMÖ ist ein entzündliches und lymphoplasmozytisches Infiltrat. Die osteoklastische Aktivität bewirkt eine subchondrale Resorption und eine periartikuläre Osteopenie sowie subchondrale zystische Veränderungen und Erosionen des angrenzenden Kortex [13]. McQueen et al. zeigten, dass dies typische Zeichen der RA sind [14]. Andere rheumatologische Erkrankungen, wie z. B. die Gicht, haben einen unterschiedlichen Pathomechanismus und sind nicht mit einer Osteopenie assoziiert.

### Synovitis

Die Synovitis der Hüfte zeigt sich als Gelenkkapselverdickung mit Ödem und Synovialzotten und kann mit einem heterogenen Erguss einhergehen. In selteneren Fällen ist die Synovitis auch entlang des Ligamentum femoris capitis zu sehen. Eine fokale Form der Synovitis kann als **pigmentierte villonoduläre Synovitis (PVNS)**

Pigmentierte villonoduläre Synovitis (PVNS)

fehlinterpretiert werden. In unklaren Fällen ist die intravenöse KM-Gabe hilfreich, um die Aktivität der Synovitis zu beurteilen und zwischen Gelenkerguss und Synovitis zu unterscheiden. Dies kann das Management und das Outcome des Patienten beeinflussen, da die aktive Synovitis bei der RA und der Gicht mit dem Knorpelschaden korreliert [14]. Vergleichbar mit dem Frozen-shoulder-Syndrom, korreliert die Entzündung der Gelenkkapsel mit einer eingeschränkten Beweglichkeit und diffusem dumpfen Gelenkschmerz. Intraartikuläre Kortisoninjektionen können den Schmerz lindern und die entzündliche Aktivität reduzieren.

### Bursitis

Unterschiedliche Schleimbeutel (Bursae) der Hüfte können im Rahmen der rheumatologischen Erkrankungen mitbeteiligt sein. Die größte Bursa des Körpers ist die **Iliopsoas-Bursa**

**Figure 8:**
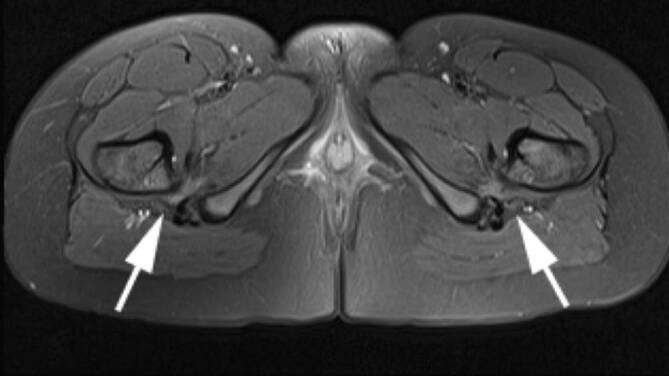

Iliopsoas-Bursa,

die bei etwa 15 % der Bevölkerung mit dem Hüftgelenk kommuniziert. In der klinischen Untersuchung ist die Iliopsoas-Bursitis nicht eindeutig von anderen Pathologien wie ischiogluteale Bursitis, Ursprungstendinose der ischiokruralen Muskulatur oder vom ischiofemoralen Impingement zu unterscheiden (Abb. 8). Die **peritrochantäre Bursitis**

Peritrochantäre Bursitis

tritt nicht nur durch Überbelastung, sondern auch bei der Spondylarthritis (SpA) und der RA auf ([15]; Abb. 7). Die sonographisch unterstützten Kortisoninjektionen in die Bursa sind hilfreich, um die lokale Entzündung einzudämmen [16]. Ein wesentlicher Vorteil der lokalen Injektion ist die niedrigere Rate von Nebenwirkungen im Vergleich zur systematischen Kortisongabe.

### Enthesitis

Die Enthesitis zeigt sich in der MRT mit einer Hyperintensität auf den T2w- Sequenzen an der Insertion von Sehnen bzw. Bändern oder an der Gelenkkapsel und ist häufig mit einem angrenzenden KMÖ assoziiert. Laut OMERACT (**Outcome Measures in Rheumatology**

**Figure 9:**
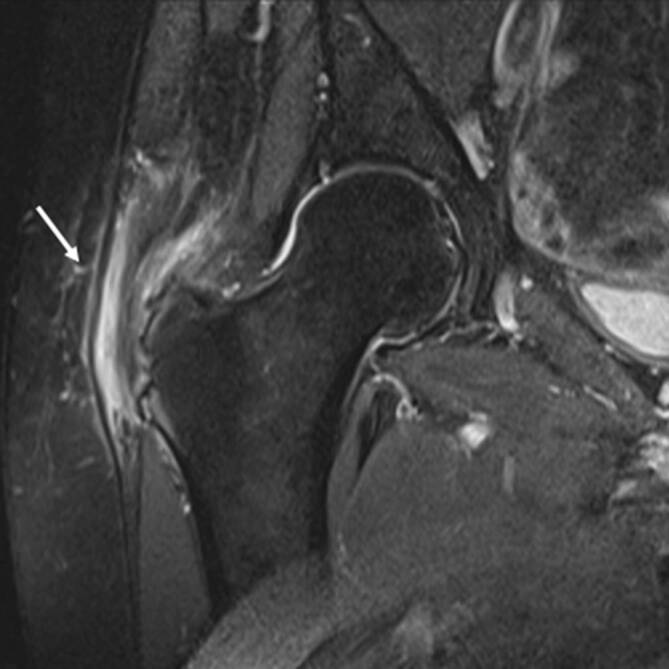

Outcome Measures in Rheumatology (OMERACT))

sollten entweder eine STIR- bzw. T2w-fs-Sequenz oder KM-unterstützte T1w-fs-Sequenzen für den Nachweise einer aktiven Entzündung an der Enthese verwendet werden ([17]; Abb. 9).

### Knorpel

Quantitative MRT-Techniken wie **dGMERIC**

dGMERIC

(„delayed gadolinium-enhanced MRI [magnetic resonance imaging] of the cartilage“), **„T2 mapping“**

„T2 mapping“,

**„T1ρ sodium MRI“**

„T1ρ sodium MRI“

und Messungen des Knorpelvolumens oder der Dicke ermöglichen den Nachweis von frühem Knorpelschaden, sind aber meist Gegenstand wissenschaftlicher Untersuchungen [18]. Diesen Techniken liegt die Messung des Verhältnisses zwischen Wasser und Proteoglykanen oder der Anteil von **Glykosaminoglykanen**

Glykosaminoglykane

(GAG) zugrunde. Im fortgeschrittenen Stadium des Knorpelschadens verschmälert sich der Knorpel der gesamten Gelenkfläche des Femurkopfes und des Azetabulums. Im Gegensatz dazu wird bei der Degeneration zuerst der Knorpel der gewichtbelasteten Gelenkanteile zerstört. Da die Bewegungseinschränkung der Gelenke stärker mit dem Knorpelschaden als mit der Knochendestruktion assoziiert ist, wird derzeit dem Knorpel bezüglich Quantität und Qualität viel Beachtung geschenkt [19].

### Erosionen

Subchondrale Erosionen erscheinen in der MRT als fokale T2-hyperintense Läsionen, die mit dem Knorpelbelag verbunden sind und in der Tiefe von einem hypointensen Saum (Sklerosesaum) umgeben sind, mit einem ähnlichen Signal wie die Kortikalis (subchondrale Platte). Im fortgeschrittenen Stadium der Hüftentzündung nehmen die Erosionen an Größe zu und resultieren in einem Kollaps des Hüftkopfs (Abb. 2) mit Irregularitäten am Azetabulum. In der Projektionsradiographie zeigt sich die Gelenkdestruktion. Ohne Anamnese oder Voruntersuchungen ist die entzündliche Destruktion häufig schwierig von einer primären Osteoarthrose oder avaskulären Osteonekrose (AVN) zu unterscheiden (Abb. 4 und 10).

**Figure 10:**
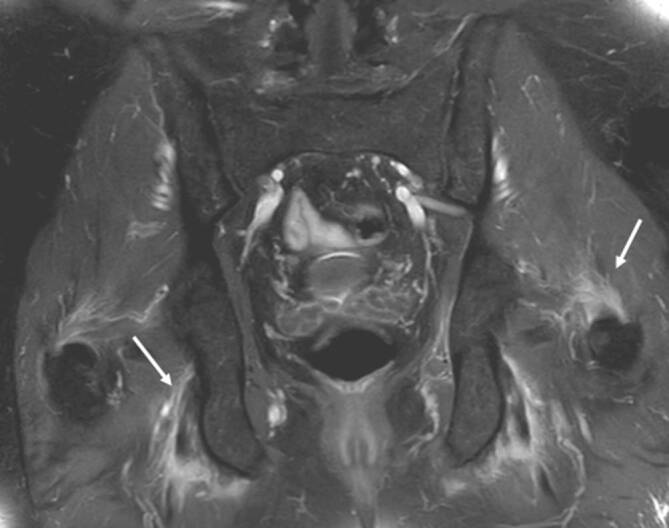

Bei der RA entstehen die Erosionen an der Hüfte zuerst an der Gelenkkapselinsertion, was analog zu den Veränderungen an der Hand ist: Die Erosionen an den kleinen Fingergelenken entwickeln sich zuerst an den nicht knorpelüberzogenen Gelenkanteilen, die auch „bare areas“ genannt werden.

## Intervention

Die **intraartikuläre Kortisoninjektion**

Intraartikuläre Kortisoninjektion

reduziert die Schmerzen und ist sinnvoll bei Synovitis, die sowohl die Gelenkkapsel als auch das Ligamentum femoris capitis betreffen kann. Jedoch auch intraartikuläre Pathologien (z. B. Labrumdegeneration) sprechen sehr gut auf die Kortisoninjektion an. Eine Studie von Pochon et al. zeigte, dass Knorpeldefekte und Knochenmarkveränderungen kein wesentliches Therapieansprechen zeigen [20]. Eine absolute Kontraindikation für die intraartikuläre Injektion ist eine Infektion der Hüfte, die unbedingt vor einer geplanten Injektion ausgeschlossen werden sollte.

Die sonographisch unterstützte Kortisoninjektion in die Bursa peritrochanterica oder in eine der subglutealen Bursae zeigt kurzfristig über einen Zeitraum von 3 bis 6 Monaten eine deutliche Verbesserung der Schmerzsymptomatik gegenüber dem konservativ behandelten Patientenkollektiv. Nach 12 Monaten konnten keine wesentlichen Unterschiede nachgewiesen werden [16].

## Unterschiedliche entzündliche Pathologien der Hüfte

### Ankylosierende Spondylitis

In zahlreichen Studien konnten 3 wichtige Risikofaktoren ermittelt werden, die zu einer vermehrten Beteiligung des Hüftgelenks im Rahmen der AS führen [21]:

1. frühzeitiger Krankheitsbeginn bereits im jugendlichen Alter,
2. fortgeschrittene axiale Beteiligung mit radiographischen Veränderungen an der Wirbelsäule und den SIG [22],
3. Enthesitis.

Die Arthritis der Hüfte (und seltener der Schulter) kommt bei etwa 50 % der Patienten mit AS vor. Klinisch zeigen sich eine Morgensteifigkeit und eingeschränkte Beweglichkeit, insbesondere in der Flexion. Der Gelenkschaden manifestiert sich durch Gelenkspaltverschmälerung, Osteoporose und im Endstadium durch eine **Ankylose**

Ankylose

**Figure 11:**
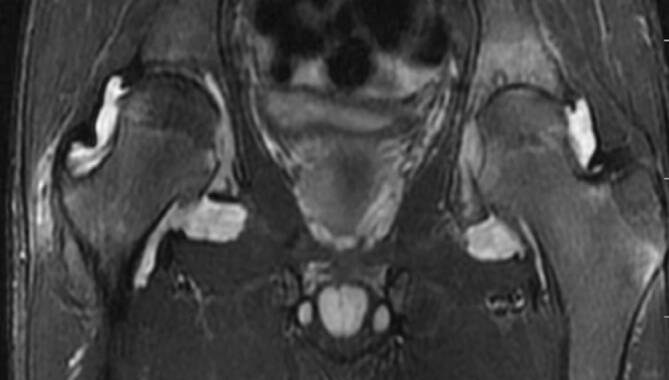

[23]. Im Gegensatz zur sonstigen osteoproliferativen Komponente der Spondylarthritis steht die subchondrale Entzündung mit ausgiebigen Erosionen an der Hüfte im Vordergrund, was zu einer raschen Gelenkdestruktion führt. Schon bei jungen Patienten ist der frühzeitige Gelenkersatz die einzige erfolgreiche Therapiemöglichkeit. Für Patienten mit Verdacht auf SpA, deren SIG in der MRT untersucht werden, empfehlen wir, das Untersuchungsfeld („field of view“, FOV) in der paraaxialen STIR/PD-fs-Sequenz zu vergrößern, damit auch die Hüftgelenke abgebildet und beurteilt werden können (Abb. 9). Die Beteiligung der Hüfte ist bei der AS häufig und wird zumeist in der MRT entdeckt, weshalb auch ein spezielles Scoring-System für die Hüftentzündung bei der SpA eingeführt wurde [24]. Huang et al. zeigten, dass bei Patienten mit AS eine Beteiligung der Hüfte mit der MRT in 74,1 % und mit der Projektionsradiographie nur in 20,7 % der Fälle gesehen wurde. Von diesen Patienten hatten lediglich 30,2 % klinische Symptome [6]. Der Gelenkerguss wird ausreichend mit der Sonographie beurteilt. Allerdings ist die MRT zielführend, um zwischen einem einfachen Gelenkerguss und einer Entzündung der Hüfte mit KMÖ zu unterscheiden (Abb. 11). Die frühzeitige Diagnose der Hüftbeteiligung ist von besonderer Bedeutung, da sie mit einem höheren Risiko einer ausgeprägten axialen Erkrankung der SpA einhergeht [23, 25]. Im Endstadium der AS sind in der Projektionsradiographie Erosionen und Deformitäten beider Hüften, ein erheblicher Knochendichteverlust und enthesopathische Veränderungen am Beckenring, eine Ankylose der SIG sowie Syndesmophyten der Lendenwirbelsäule (LWS) nachweisbar (Abb. 2). Die peritrochantäre Bursitis kann bei der SpA vorkommen und wird am besten in der Sonographie oder in den T2w-fs-Sequenzen der MRT dargestellt (Abb. 7). Die Bursitis weist einen lateralen Hüftschmerz auf, der seitlich in das Kniegelenk ausstrahlen kann und druckschmerzhaft ist. Die entzündlich-rheumatisch verursachte Bursitis sollte gegenüber der Tendinose mit Teilruptur der Gluteus-medius- und -minimus-Sehnen-Insertion am Trochantor major oder gegenüber der funktionellen Störung des Bewegungsapparats mit myofaszialen Schmerzen, durch ein Friktionsyndrom des iliotibialen Bands über dem posterolateralen Anteil des Trochanter major, abgegrenzt werden.

### Psoriasisarthritis

Die Hüfte ist bei der Psoriasisarthritis (PsA) nur selten beteiligt und tritt dann häufiger bei jungen Patienten mit axialer Arthritis auf [26]. Die Entzündung der beiden Hüften verläuft üblicherweise progressiv, und laut einer Studie von Michet benötigen 50 % der Patienten innerhalb von 5 Jahren nach Erstdiagnose einen Hüftgelenkersatz [26]. **Marginale Osteophyten**

Marginale Osteophyten

und **konzentrische (gleichmäßige) Gelenkspaltverschmälerungen**

Konzentrische Gelenkspaltverschmälerungen

sind charakteristisch für die primäre Hüftentzündung im Gegensatz zur Arthrose, bei der die gewichtbelasteten Areale eine Knorpelbelagverschmälerung aufweisen. Ein gleichzeitiges Auftreten der Sakroiliitis ist hilfreich, um die PsA der Hüfte von der Coxarthrose zu unterscheiden.

### Juvenile idiopathische Arthritis

Die juvenile idiopathische Arthritis (JIA; ehemals: juvenile rheumatoide Arthritis) wird seit 2001 von der International League of Associations for Rheumatology (ILAR) in 7 Subtypen unterteilt. Die Kriterien dafür sind:

- gleichzeitig auftretende systemische Entzündung mit Fieber und Hautveränderungen,
- Anzahl der betroffenen Gelenke (< oder > 5 Gelenke),
- mit oder ohne Rheumafaktor,
- Vorkommen von PsA oder Enthesitis.

**Figure 12:**
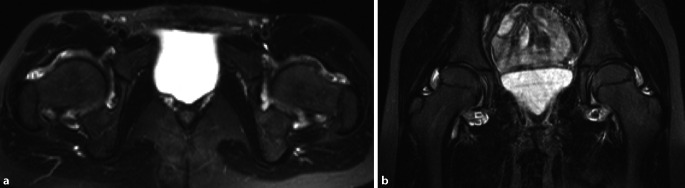

Bei der JIA ist am häufigsten das Kniegelenk betroffen, jedoch kann auch das Hüftgelenk in Abhängigkeit von der Krankheitsdauer in etwa 7–19 % der Fälle entzündet sein [27]. Die Synovitis ist wahrscheinlich das zuverlässigste Zeichen in der MRT, um die JIA von anderen Formen einer Arthritis bei Kindern zu differenzieren ([28]; Abb. 12a, b). Im Gegensatz zur Erwachsenenform der RA sind bei einer progressiv-destruierenden Form der JIA bei 30–50 % der Kinder die Hüftgelenke betroffen [29]. Die Folgen haben einen erheblichen Einfluss auf die zukünftige körperliche Behinderung, da die Hüftgelenke entscheidend für die Mobilität sind. Laut Kirkhus et al. ist die Synovitis bei JIA häufiger nicht aktiv (nicht KM-anreichernd), eher fibrös und erscheinen daher mit dunklem MRT-Signal und irregulärer Dicke im Vergleich zur infektiösen Arthritis [30]. Nistala et al. bestätigen, dass die **Erythrozytensedimentationsrate**

Erythrozytensedimentationsrate

(ESR) ein prognostischer Faktor für die aktive Hüftentzündung der JIA in der MRT ist [12]. Eine erhöhte ESR ist spezifisch für die Hüftentzündung, jedoch kann auch bei normaler ESR eine Hüftentzündung vorkommen.

### Rheumatoide Arthritis

**Figure 13:**
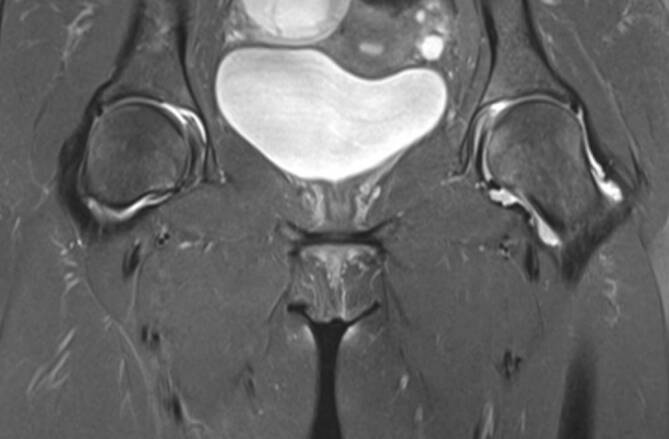

**Figure 14:**
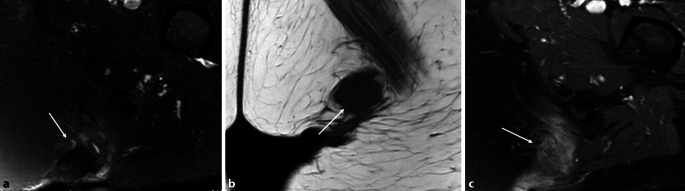

Die RA ist häufiger bei Frauen und kann in jedem Alter, auch bei Kindern als JIA, auftreten. Im Gegensatz zur Osteoarthritis, die nur einseitig vorkommen kann, ist ein bilateraler Befall typisch für die RA. Die MRT zeigt frühe Veränderungen wie KMÖ, Gelenkergüsse und Synovitis (Abb. 13). Selten können auch rheumatoide Knoten in der periartikulären Muskulatur auftreten (Abb. 14). Neben der häufiger auftretenden **peritrochantären Bursa**

peritrochantäre Bursa

kann auch die Iliopsoas-Bursitis Hüftschmerzen bei der RA verursachen, was erfolgreich mit Kortisoninjektionen behandelt wird [31]. Der Hüftschmerz bei RA zeichnet sich durch Schmerzen und Morgensteifigkeit im Oberschenkel und in der Leiste aus. Die (gelenknahe) Osteoporose ist eine der Hauptkomplikationen der RA und wird durch die Stimulation von Osteoklasten verursacht. Die Osteoklasten werden durch die Entzündungskaskade (Interleukin[IL]-6) und durch die Kortisontherapie stimuliert [32]. Aus diesem Grund sollte bei rheumatologischen Patienten unter Kortisontherapie die Knochenmineraldichte engmaschig kontrolliert werden und rechtzeitig eine notwendige Therapie mit Vitamin D und Bisphosphonaten eingeleitet werden, um osteoporotische Frakturen zu vermeiden.

#### Merke

Der Knorpelbelag ist bei der rheumatoiden Arthritis diffus verschmälert, während bei der Coxarthrose der Knorpel in den gewichttragenden Anteilen primär verschmälert ist.

### Polymyalgia rheumatica

Die EULAR/ACR(American College of Rheumatology)-Klassifikation aus 2012 für die Polymyalgia rheumatica (PMR) [37] schließt einen neu aufgetretenen Hüftschmerz bei Patienten über 50 Jahre mit beidseitigem Schulterschmerz, Morgensteifigkeit für mehr als 45 min und erhöhtem **C‑reaktiven Protein**

C‑reaktives Protein

(CRP) und/oder erhöhter **Blutsenkungsgeschwindigkeit**

Blutsenkungsgeschwindigkeit

(BSG) als wichtiges Kriterium ein. Die Synovitis der Hüfte und die Bursitis peritrochanterica werden nun als gleichwertige Kriterien wie die Entzündung der Schulter angesehen. Bei älteren Menschen ist die PMR die häufigste Autoimmunerkrankung und schwierig von der RA bei spätem Auftreten im hohen Alter zu unterscheiden. Das periartikuläre Weichteilödem ist allerdings häufiger bei der PMR als bei der RA zu sehen [33]. Die PMR kann in etwa 20 % der Fälle mit einer **Riesenzellarteriitis**

Riesenzellarteriitis

assoziiert sein [34]. Die peritrochantäre Bursitis ist die häufigste Pathologie der Hüfte bei der PMR. Aufgrund der tiefen Lage der Iliopsoas-Bursitis und der Synovitis der Hüftgelenks können diese Pathologien verlässlicher mit der MRT als mit der Sonographie dargestellt werden [35].

### Kristallarthropathien

Es gibt mehrere unterschiedliche Entitäten, die unter den Kristallarthropathien zusammengefasst werden [36]. Die 3 wichtigsten davon sind:

- Chondrokalzinose,
- HADD („hydroxyapatite deposition disease“),
- Gicht.

**Figure 15:**
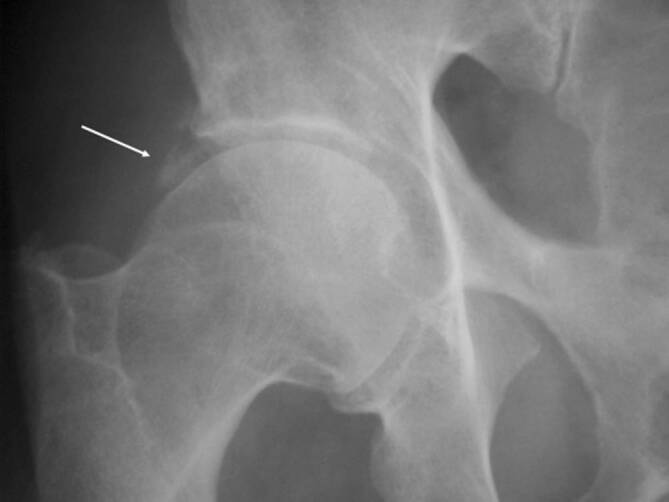

Die Chondrokalzinose wird auch Pseudogicht oder CPPD („calcium pyrophosphate dehydrate crystal deposition disease“) genannt. Bei der CPPD sind die Kalziumpyrophosphatablagerungen innerhalb des Knorpels, in den Menisci oder im triangulären fibrokartilaginären Komplex zu finden (Abb. 15). Bei der HADD sind sowohl die periartikulären als auch die intraartikulären Regionen betroffen. Die Projektionsradiographie ist die beste bildgebende Methode, um die runden oder ovalen Verkalkungen in den paraartikulären Sehnen, in den Bursae oder in der Gelenkkapsel nachzuweisen. Zumeist ist die Schulter betroffen, allerdings kann jedes Gelenk, auch die Hüfte, involviert sein. Die Gicht befällt nur selten die Hüfte und ist dann aber durch Synovitis, Gelenkerguss und ausgestanzte juxtaartikuläre Erosionen gekennzeichnet. Die Gichtkristallablagerungen können im Erguss als Sternenhimmel erscheinen. Häufig finden sich die Gichtkristallablagerungen auf der Oberfläche des Knorpels und erscheinen sonographisch als **Doppellinienzeichen**

Doppellinienzeichen

(Reflexion der Kristalle und des subchondralen Knochens).

Patienten mit Nierenversagen und langwieriger Hämodialyse können das Amyloid **β2-Mikroglobulin**

β2-Mikroglobulin

in den Gelenken ansammeln, was in der Folge zur Gelenkdestruktion im Rahmen der Amyloidarthropathie führt. Die Wirbelsäule ist prädisponiert, allerdings werden auch Hüfte, Schultern und Karpalknochen befallen. In der Projektionsradiographie sind typischerweise eine geringe periartikuläre Osteoporose, große subchondrale zystische Läsionen mit gut begrenzten sklerotischen Rändern und eine juxtaartikuläre Weichteilschwellung zu sehen. Jedoch bleibt im Gegensatz zur Arthrose oder zu anderen entzündlichen Erkrankungen der Gelenkspalt üblicherweise erhalten. Die Amyloidablagerungen sind in der MRT von niedrigem oder intermediärem T1w- und T2w-Signal und weisen auf Gradientenechosequenzen Suszeptibilitätsartefakte auf.

#### Merke

Die Kristallablagerungen der Chondrokalzinose sind innerhalb des Knorpels, in den Menisci oder im Discus zu finden, während bei der Gicht die Ablagerungen auf der Oberfläche des Knorpels nachweisbar sind.

## Zusammenfassung

Der entzündliche Hüftschmerz ist häufig ein unspezifischer Schmerz, der jedoch eine weitere bildgebende Abklärung erfordert. Neben der Hüftbeteiligung im Rahmen der Spondylarthritis, der RA oder der JIA wurde seit 2012 bei der PMR auch der Hüftschmerz in die Klassifikationskriterien eingeschlossen. Diese entzündlichen Formen des Hüftschmerzes sollten von den Kristallarthropathien wie Gicht, HADD und CPPD abgegrenzt werden.

## Fazit für die Praxis

- Sonographischer Gelenkerguss und Synovitis der Hüfte sind unspezifisch und erfordern häufig eine weitere Abklärung mittels Magnetresonanztomographie (MRT).
- Eine gleichmäßige Verschmälerung des Gelenkspalts weist auf eine rheumatische Ursache hin, während die Degeneration den Gelenkspalt im gewichttragenden Anteil verschmälert.
- Die peritrochantäre Bursitis tritt häufig bei der rheumatoiden Arthritis und der ankylosierenden Spondylitis auf und sollte von der mechanisch-funktionell verursachten peritrochantären Bursitis, die häufig mit Beteiligung der Gluteussehneninsertion einhergeht, abgegrenzt werden.

## CME-Fragebogen

Welches ist die erste bildgebende Modalität zur Abklärung des Hüftschmerzes?

Hüftsonographie

Magnetresonanztomographie

Computertomographie

Projektionsradiographie

Szintigraphie

Auf welche MRT(Magnetresonanztomographie)-Sequenz kann unter *keinen* Umständen beim entzündlichen Hüftschmerz verzichtet werden?

STIR („short tau inversion recovery“)

T2-Gradientenechosequenz

T1-fettunterdrückte Sequenz

Hemoepi-Sequenz

Kontrastmittelunterstützte T1-gewichtete Sequenz

Was ist *nicht* typisch für die Enthesitis?

Knochenmarködem an der Sehneninsertion

T2-Hyperintensität am Bandansatz

Sehnenruptur

Entzündliche Veränderungen an der Gelenkkapsel

Paratendinitis

Welche Veränderung am Hüftgelenk wird *nicht* bei der Spondylarthritis gesehen?

Synovitis

Bursitis

Erosionen

Enthesitis

Weichteilverkalkungen

Was tritt bei der Gicht in der Bildgebung *nicht* auf?

Gelenknahe Osteopenie

Ausgestanzte juxtaartikuläre Erosionen

Synovitis

Doppellinienzeichen durch Kristallablagerungen

Gelenkerguss

Was ist eine absolute Kontraindikation für die intraartikuläre Kortisoninjektion?

Systemische Kollagenose

Bakterielle Entzündung des Gelenks

Rheumatoide Arthritis

Vorliegender Knorpeldefekt

Synovitis

Wo sind *keine* Verkalkungen bei der Chondrokalzinose zu finden?

Knorpel

Labrum

Bursa

Knochen

Gelenkkapsel

Was ist *keine* Spätkomplikation der rheumatoiden Arthritis der Hüfte?

Septische Arthritis

Avaskuläre Nekrose

Osteoarthrose

Chondromalazie

Osteoporotische Fraktur

Warum sollte das Untersuchungsfeld in der Magnetresonanztomographie der Sakroiliakalgelenke bei Verdacht auf Spondylarthritis vergrößert werden?

Darstellung der Hüftgelenke.

Vergleich mit der kontralateralen Hüfte.

Darstellung des Pudendusnervs im Alcock-Kanal.

Nebennierenadenome sind häufig.

Darstellung der Hamstring-Muskelatrophie.

Was ist die häufigste Pathologie an der Hüfte bei der Polymyalgia rheumatica?

Morgensteifigkeit

Periartikuläres Weichteilödem

Iliopsoas-Bursitis

Synovitis Hüftgelenk

Peritrochantäre Bursitis
